# Supplementary material for: Micronutrient optimization for tissue engineered articular cartilage production of type II collagen
Source: Front Bioeng Biotechnol. 2023 Jun 6;11:1179332. doi: 10.3389/fbioe.2023.1179332 (PMC10280293; doi:10.3389/fbioe.2023.1179332)
Supplement: Supplementary file 1 [file DataSheet2.PDF]

Term	Conditions (µg/ml)				
	12	25	52	72	89
**Lin**	6.6E-09	3.3E-08	1.1E-08	6.8E-09	5.6E-09
**Cr**	3.9E-04	6.8E-04	4.9E-05	6.6E-04	1.1E-03
**Co**	5.8E-06	1.2E-04	1.3E-06	1.8E-06	5.5E-06
**Cu**	2.1E-01	2.2E-08	6.7E-01	4.6E-02	5.0E-02
**I**	9.0E-02	1.5E-02	9.2E-02	9.2E-02	9.0E-02
**Mn**	1.2E-02	4.5E-05	4.7E-04	8.6E-03	3.8E-03
**Mo**	1.9E-03	1.5E-03	2.0E-03	2.0E-03	1.1E-03
**Thy**	2.5E-02	2.6E-02	2.4E-02	2.4E-02	2.4E-02
**Vit A**	8.4E-11	3.0E-11	2.5E-11	1.0E-10	9.8E-11
**Vit B12**	9.1E-07	6.3E-12	1.3E-06	9.1E-07	9.7E-07
**Vit B7**	2.8E-03	2.3E-03	3.0E-03	9.3E-05	1.9E-03
**Vit D**	7.2E-06	9.9E-10	1.7E-05	8.7E-08	8.7E-08
**Vit E**	1.5E-08	5.9E+01	1.6E-08	1.5E-08	2.0E-08
**Vit K**	4.7E-06	5.0E-12	5.6E-06	2.8E-05	9.0E-06
**Zn**	3.6E-03	2.3E+00	1.8E-03	4.4E-03	1.8E-03
Desirability	*0.835*	*0.595*	*0.831*	*0.829*	*0.827*

Supplemental Table 2: Optimal predicted combinations of vitamins and minerals as derived by DoE.

a.

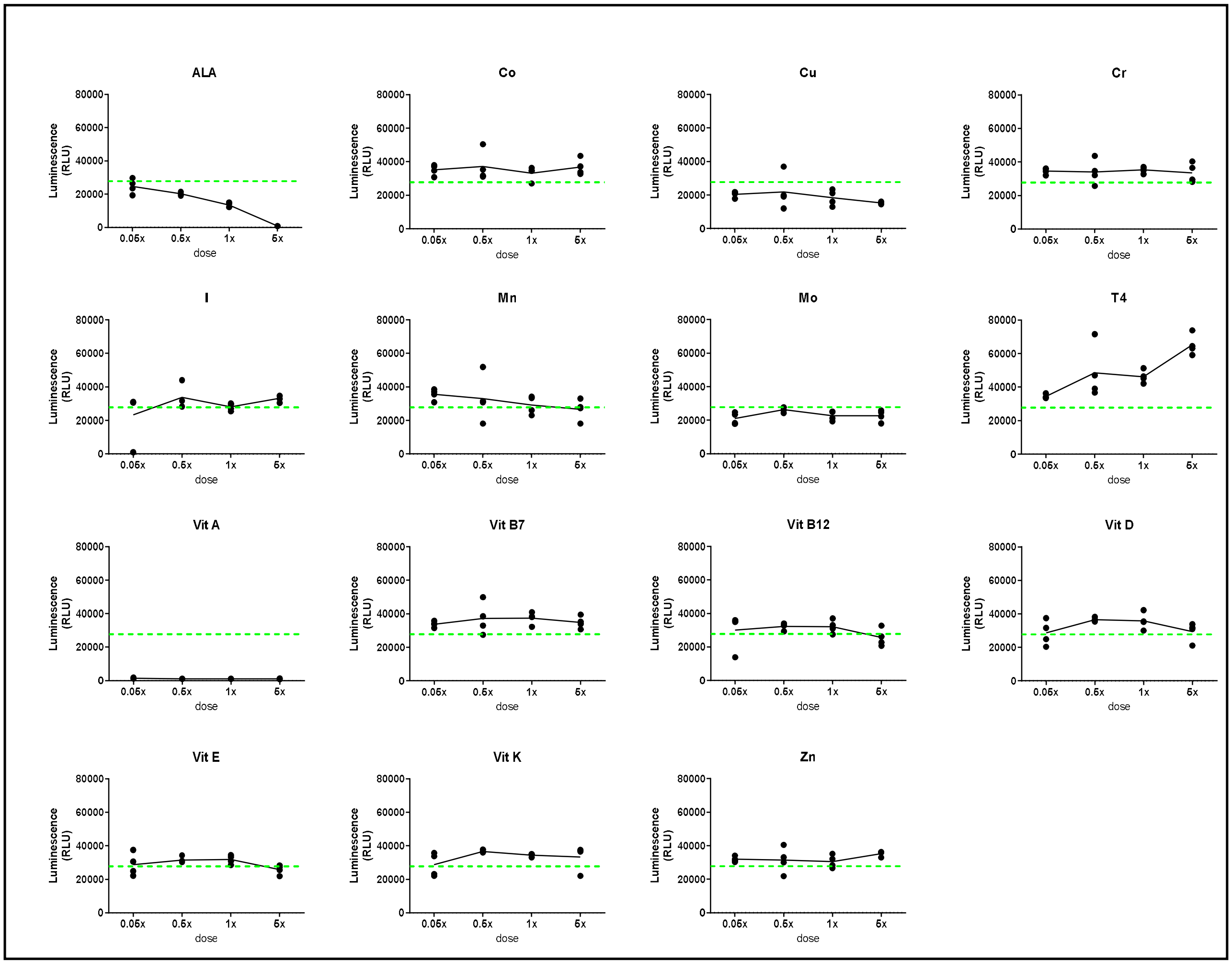

b.

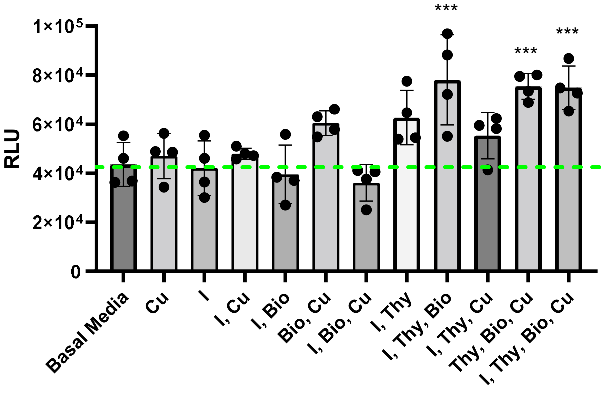

Supplemental Fig 1: a, b Primary COL2A1-GLuc rabbit chondrocytes were seeded in aggregate culture in basal chondrogenic media supplemented with different concentrations of a single vitamin or mineral **(a)** or combinations **(b)**. Media was assessed for luminescence and results are shown for day 21. Individual values for 4 replicates are shown with green dashed line indicating basal media mean. Error bars **(b)** indicate standard deviation and *** indicate $p < 0.001$ vs. basal media control.

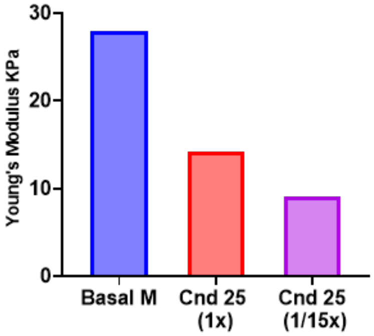

Supplemental Fig 2: COL2A1-GLuc primary rabbit chondrocytes were cultured in custom in house bioreactors. At day 22, biopsy punches of engineered sheet were assessed via compression testing and young's equilibrium modulus calculated.

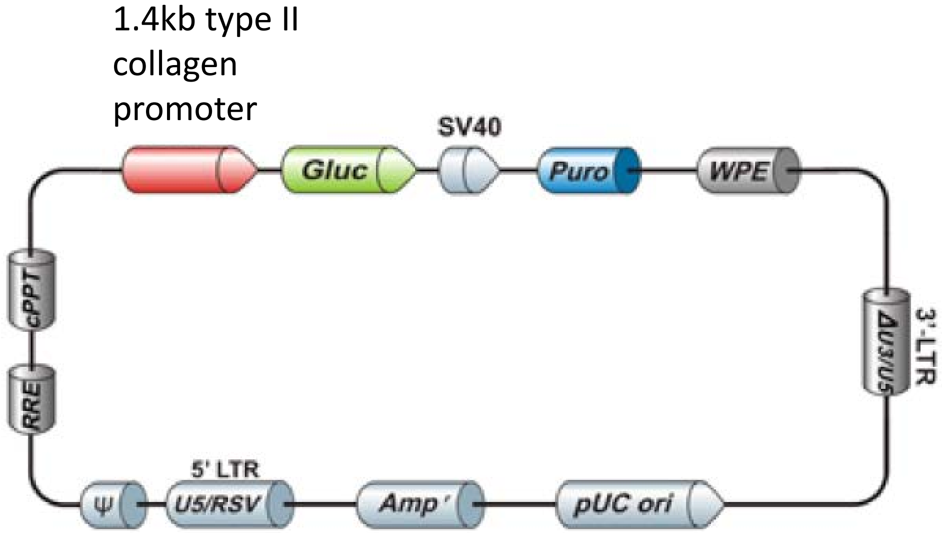

Supplemental Fig 3: COL2A1-GLuc plasmid map
COL2A1-GLuc (HPRM22364LvPG02; Genecopoeia) has a 1.4kb promoter-driven *Gaussia* luciferase containing 1245 bp upstream of the transcriptional start site and 180 bp downstream. It also contains a puromycin (Puro) selection cassette for mammalian cell selection and an ampicillin (Amp) selection cassette for bacterial selection.

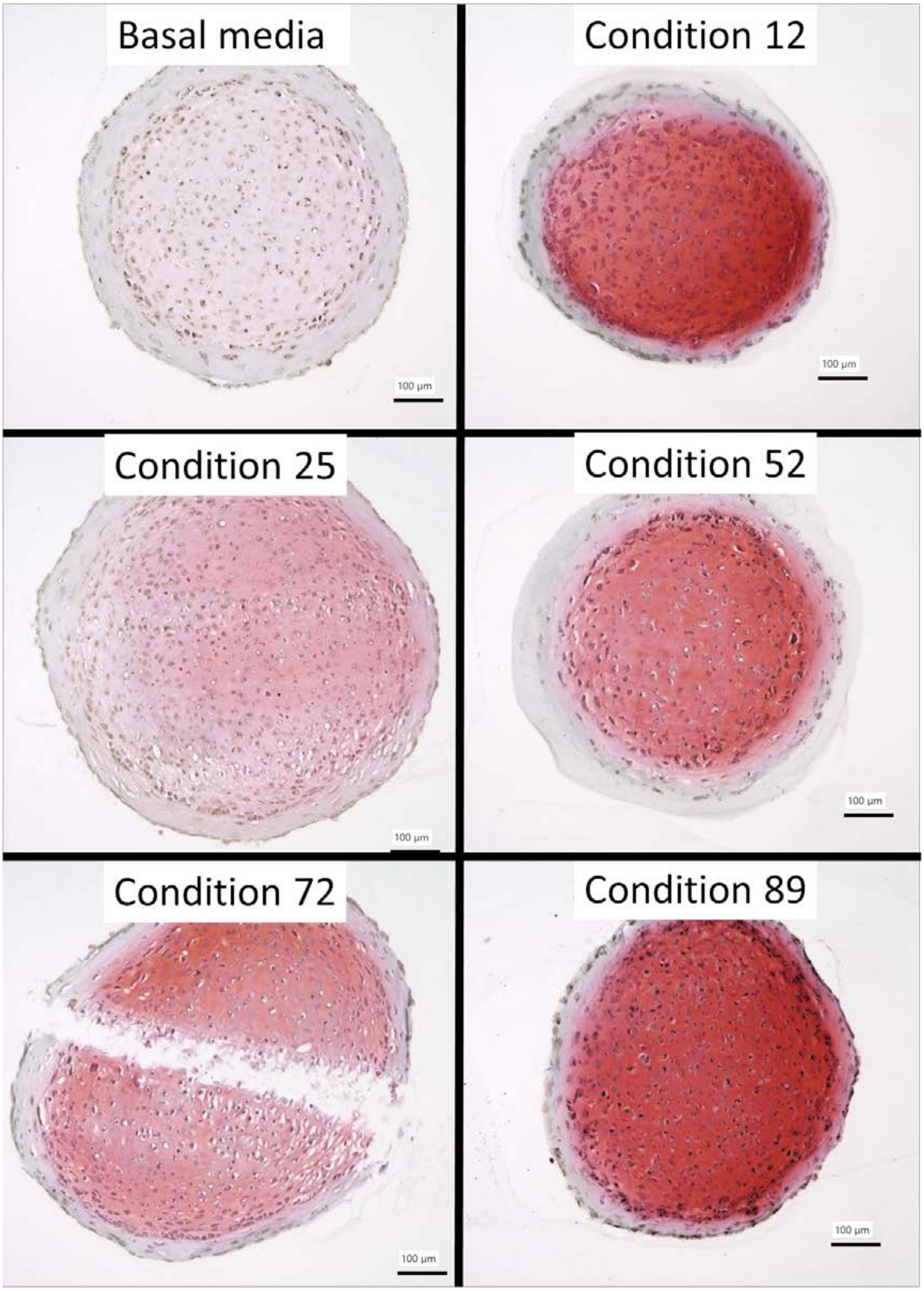

Supplemental Fig 4: Glycosaminoglycan (GAG) staining of aggregates
Safranin-O, fast-green and hematoxylin stained neutral buffered formalin fixed, paraffin embedded 5µm sections.
